# Diagnostic challenges in vestibular migraine—clinical differentiation from Menière’s disease and discrepancies with current classification criteria

**DOI:** 10.1007/s00415-025-13291-x

**Published:** 2025-08-05

**Authors:** Doreen Huppert, Eva Grill, Sandra Becker-Bense, Andreas Zwergal, Ralf Strobl

**Affiliations:** 1 German Center for Vertigo and Balance Disorders (DSGZ), LMU University Hospital, LMU Munich, Munich, Germany; 2 Department of Neurology, LMU University Hospital, LMU Munich, Munich, Germany; 3 Institute for Medical Information Processing, Biometrics and Epidemiology, LMU Munich, Munich, Germany

**Keywords:** Vestibular migraine, Episodic vestibular disorder, Menière’s disease, Headache, Vertigo

## Abstract

**Background:**

Vestibular migraine (VM) and Menière’s disease (MD) are spontaneous episodic vestibular syndromes and often present with overlapping features, making clinical differentiation challenging. This study aimed to (1) identify key features distinguishing VM from MD and (2) investigate discrepancies between expert diagnosis and International Classification of Vestibular Disorders (ICVD) criteria for VM.

**Methods:**

We analyzed data from patients diagnosed with VM or MD at the tertiary dizziness center of LMU Munich. Diagnostic classification was based on ICVD criteria and expert judgment. Symptoms, vestibulo-ocular reflex (VOR) function, and demographics were compared. A conditional inference tree identified key differentiators. For ‘suspected VM’ cases not meeting ICVD criteria, reasons for diagnostic discrepancy were analyzed.

**Results:**

We included 290 patients: 188 with VM and 88 with MD. VM was more common in women (72% vs. 51%) and had an earlier onset (39.6 vs. 49.9 years). MD patients had more rotational vertigo, greater caloric asymmetry, and lower VOR gains on video head impulse testing (all *p* < 0.0001). The tree identified seven key variables and achieved 86% accuracy. Sixty-six VM patients were diagnosed as ‘suspected VM’ based on expert judgment. Discrepancies were primarily due to short attack duration and atypical symptoms.

**Conclusions:**

This study identified seven clinical variables to effectively distinguish VM from MD. While VM and MD share overlapping features, diagnostic ambiguity remains common, particularly in cases not meeting ICVD criteria. Our findings support the introduction of a ‘suspected VM’ category to capture patients with atypical presentations not covered by ICVD criteria.

## Introduction

The two most common spontaneous episodic vestibular syndromes are vestibular migraine (VM) with a lifetime prevalence of 1% [1], followed by Menière’s disease (MD) [2]. VM and MD are sometimes difficult to differentiate, even for an experienced clinician after thorough history taking and clinical examination. Previously assumed differentiation criteria such as endolymphatic hydrops (EH) on contrast-enhanced high-resolution temporal bone MRI no longer provide a reliable basis for differentiation. While EH has been well-documented in MD [3–6], it recently was also found in patients with VM and other vestibular disorders [7–10].

This diagnostic ambiguity has prompted the development of standardized clinical criteria. In 2012 resp. 2015 diagnostic criteria for VM and MD were formulated with the framework of the International Classification of Vestibular Disorders (ICVD) by the International Bárány Society for Neuro-Otology [11–13] and were revised for VM in 2022 with regard to a literature update, but without essentially changing the criteria [14] (see Table 1 for an overview of the current definitions). To complicate matters, there are no reliable specific symptoms for one or the other condition, e.g., that both headache and auditory symptoms may occur in VM and MD. Further, epidemiological studies revealed a coincidence of both conditions in individual patients [15–17]; this fact even led to the proposal of a classification of ‘VM/MD overlap syndrome’ as a new clinical syndrome of patients with definite VM and definite MD [18]. This may pose uncertainties in the evaluation of the response of medical treatment and various subsequent therapeutic interventions may be required.

**Table 1:** Current definitions of vestibular migraine and Menière’s disease

Vestibular migraine	A. At least 5 episodes with vestibular symptoms of moderate or severe intensity, lasting 5 min to 72 h B. Current or previous history of migraine with or without aura according to the International Classification of Headache Disorders (ICHD) C. One or more migraine features with at least 50% of the vestibular episodes: - headache with at least two of the following characteristics: one-sided location, pulsating quality, moderate or severe pain intensity, aggravation by routine physical activity - photophobia and phonophobia - visual aura D. Not better accounted for by another vestibular or ICHD diagnosis Probable: A, B or C, and D
Menière’s disease	A. Two or more spontaneous episodes of vertigo, each lasting 20 min to 12 h B. Audiometrically documented low- to medium-frequency sensorineural hearing loss in one ear, defining the affected ear on at least one occasion before, during or after one of the episodes of vertigo C. Fluctuating aural symptoms (hearing, tinnitus, or fullness) in the affected ear D. Not better accounted for by another vestibular diagnosis Probable: A, C, and D

Adapted from Lempert et al. 2022 [14] for vestibular migraine and Lopez-Escamez et al. 2015 [13] for Menière’s disease

The current diagnostic criteria are optimized to differentiate typical presentations of MD and VM, e.g., to define homogenous cohorts for clinical trials, but may pose difficulties in daily clinical care. For example, patients presenting with symptoms of VM, but without five previous attacks, currently cannot be classified as VM.

These diagnostic uncertainties and the revision of the diagnostic guidelines without changing the diagnostic criteria prompted us to collect real-world clinical features of these two spontaneous episodic vestibular syndromes, including potentially predictive symptoms, interictal ocular motor findings, vestibular diagnostic test results and additional parameters, that may enable differentiation beyond the current strict ICVD classification. The primary objective of this study was to characterize patients with VM and MD presenting at a specialized tertiary dizziness center and to identify factors that can effectively differentiate these two conditions. The second study aim was to investigate the reasons for discrepancies in diagnostic classification of VM based on clinical expert evaluations and ICVD criteria, specifically addressing the limitations of the conventional classification into (definite) VM and probable VM in the context of daily practice in a specialized outpatient setting.

## Methods

### Study design and data sources

The patients of this cohort study were selected from DizzyReg, which is an ongoing prospective clinical patient registry at the German Center for Vertigo and Balance Disorders (DSGZ), LMU University Hospital Munich. DizzyReg collects clinical data in a standardized way to create a comprehensive clinical database of patient characteristics, symptoms, diagnostic procedures, diagnosis, therapy, and outcomes in patients with vertigo and balance disorders [19]. Specific criteria for the selection of patients are described in more detail below.

General inclusion criteria into the registry are symptoms of vertigo and dizziness, age 18 years and above, signed informed consent and sufficient knowledge of German. The study was conducted in accordance with the ethical standards outlined in the Declaration of Helsinki. Data protection clearance and approval from the institutional ethics committee were obtained (Nr. 414-15).

### Diagnosis

All patients underwent a complete neurological, neuro-ophthalmological, and neuro-otological examination administered by experienced experts in the field in accordance with the diagnostic guidelines [11, 13, 14, 20–26]. Laboratory examinations included neuro-orthoptic procedures (i.e., determination of the subjective visual vertical and fundus photography), head-impulse test with video-oculography, caloric testing, ocular motor assessment by video-oculography, pure-tone audiometry, posturography, and gait analysis when clinically necessary. All examinations were performed and analyzed according to the established valid and reliable methods and standards used worldwide.

For this study, we included all patients with either a diagnosis of MD or VM. The diagnoses were established using two approaches: first, by applying the diagnostic criteria outlined in Table 1, and second, based on the expert opinion of experienced neuro-otological professionals, who synthesized comprehensive clinical information from all examinations while fully considering the patient’s medical records. To ensure clarity, patients with mixed syndromes were excluded. Patients were classified as’suspected VM’ if they were not classified as VM based on the ICVD criteria but as VM based on expert opinion.

### Variables

To comprehensively characterize patients presenting with either VM or MD, we report variables that assess the impact of the disease on patients’ life and those that distinguish between MD and VM. The latter includes patient-specific factors such as age, gender, and lifestyle, as well as variables related to functional capacity, including laboratory test results and clinical signs and symptoms such as ear pressure or standing problems. Lifestyle factors, sociodemographic data, self-perceived symptoms and triggers, such as attack duration or time since vertigo onset, and the overall impact on daily life were collected using self-assessment questionnaires [19]. Further details on the assessed variables are provided below.

#### Lifestyle and sociodemographic factors

Age was determined based on the date of admission. Leisure-time physical activity was assessed using two separate questions regarding engagement in sports during the winter and summer months (including cycling). Responses were categorized as"inactive"(combining “no activity” and “low activity”) or"active"(including “moderate activity” and “high activity”). Smoking behavior was evaluated through questions on the average number of cigarettes smoked per day, smoking frequency (regular vs. occasional), and the year of smoking cessation, if applicable.

#### Self-reported clinical signs and symptoms

During the consultation, patients completed a standardized self-report questionnaire to document their most prominent symptoms, along with the duration, frequency, and nature of their vertigo episodes. Self-reported symptoms encompassed a broad range of vestibular, visual, and somatic complaints, including rotational vertigo, imbalance, dizziness, blurred or double vision, persistent vertigo, nausea and vomiting, gait and postural instability, ocular motor disturbances, visual impairment, headache, photophobia, paresthesia, auditory symptoms (e.g., hearing loss, ear pressure, tinnitus), as well as neck pain and sensations of head pressure. To describe the nature of headache in patients with VM and to investigate its influence on discrepancies between ICVD-based and expert diagnoses, relevant information was extracted from patient records and evaluated by experienced clinicians. Headache type was classified as either pressure-like or pain-like, intensity was categorized as mild, moderate, or severe, and localization was recorded as frontal or occipital.

#### Ocular motor findings

Ocular motor findings were systematically assessed during clinical examination, typically during the asymptomatic phase at the time of presentation. The examination included spontaneous nystagmus, provocation-induced nystagmus elicited by head-shaking, gaze-evoked nystagmus, smooth pursuit, upbeat and downbeat nystagmus, and fixation-suppressed nystagmus. In addition, fundus inspection and deviations of the subjective visual vertical (SVV) were recorded. All assessments were performed by experienced clinicians and interpreted in the context of central versus peripheral vestibular dysfunction. Findings were documented as part of the standardized clinical record.

#### Apparative test variables

##### Video head impulse test

Video head impulse testing (vHIT) was performed with the EyeSeeCam® system [27]. For each participant the gain value of the vestibulo-ocular reflex was measured and the presence of refixation saccades were noted. We summarized the result of the vHIT as the median gain. Median gain was classified as “pathological” if the value on either the left or right side was below 0.7, and as “normal” otherwise. In addition, gain loss was categorized as “unilateral” if the threshold was missed on one side, as “bilateral” if both sides were below the threshold, and as “normal” if neither side showed a pathological value.

##### Side difference in caloric testing

Caloric testing comprises of vestibular stimulation of the horizontal semicircular canals on both sides with hot and cold water. For each stimulation the slow phase velocity (SPV; degrees per second) was recorded for both conditions. The results were then analyzed as the side difference by dividing the absolute difference between the responses from the right and left ears by the sum of all absolute response values following Jongkees formula [28]. Side difference was categorized as “pathological” for values above 25% and “normal” otherwise.

##### Caloric–vHIT dissociation

Caloric–vHIT dissociation is a recently proposed diagnostic marker, describing a mismatch between low-frequency vestibular function assessed by caloric testing and high-frequency function assessed by the vHIT. This pattern has been reported as a typical finding in Menière’s disease and may aid in differentiating it from vestibular migraine and other vestibular disorders [29]. In the present study, we adopted this concept as the presence of a pathological caloric side difference (> 25%) in combination with a normal horizontal vHIT gain (> 0.7).

##### Audiometric data

Audiometric data were retrieved from patient records, including both in-house pure-tone audiometry and externally provided medical reports. Pure-tone audiometry was performed in a standardized manner at 1, 2, 4, and 8 kHz, with a hearing threshold of ≥ 20 dB HL considered pathological. As external documentation also relied on qualitative descriptors (e.g., “normal hearing,” “severe hearing loss”) without quantitative thresholds, an uniform application of standardized audiometric classification criteria was not feasible.

#### Perceived impact on patients’ life

Perceived impact of the disease on patients’ life was assessed with the Dizziness Handicap Inventory (DHI). The DHI is a widely used measure to assess self-perceived limitations posed by vertigo and dizziness [30]. Twenty-five questions are used to evaluate functional, physical and emotional aspects of disability because of vertigo or dizziness. The total score ranging from 0 to 100 is derived from the sum total of responses (0 = No, 2 = sometimes, 4 = Yes) with higher scores indicating worse states. The DHI has been shown to contain three different dimensions: emotional (9 items), physical (7 items), and functional (9 items) impairment.

### Statistical analyses

Descriptive statistics were summarized using mean and standard deviation for continuous variables and absolute and relative frequencies for categorical variables. We tested group differences using two-sided independent samples t-tests for continuous variables and Chi-squared tests for categorical variables. Group comparisons were made between the VM and MD groups, as well as between patients with discordant diagnoses based on clinician expert evaluation and the ICVD criteria, i.e., misclassified patients.

#### Reasons of disagreement

Specifically, patients with suspected vestibular migraine were of particular interest, as exclusive reliance on ICVD criteria may lead to underdiagnosis or missed cases. To better understand the factors contributing to diagnostic discrepancies, we systematically analyzed the clinical profiles and underlying reasoning in cases classified as suspected VM. The reasons for diagnostic disagreement were initially recorded as free-text comments by experienced clinicians and subsequently categorized and synthesized to identify the most relevant contributing factors.

#### Conditional inference tree

To identify variables that differentiated MD and VM we used machine learning, namely conditional inference trees (CIT) [31, 32].

CIT results in a visually intuitive tree structure that mimics human decision-making and is straightforward to interpret. Briefly, the algorithm splits the dataset into smaller, more homogeneous subsets based on a defined outcome—in this study, VM or MD as based on expert opinion. This process is visualized as an inverted tree, with each node representing a differentiating variable and each branch representing a split of the sample. After each split, the subgroups are more homogeneous regarding the outcome, i.e., they contain a smaller percentage of patients with a VM or a MD diagnosis. The splitting process continues until each branch ends in a terminal node that is as homogeneous as possible regarding the diagnosis. Each terminal node is assigned to the class most frequently occurring within it. Without constraints, the tree may grow excessively, perfectly classifying the training data but performing poorly on new data due to overfitting. The conditional inference framework applies statistical significance thresholds to control tree complexity, i.e., by applying a statistical test for the global null hypothesis of independence between any of the input variables and the response. At each step the variable with the strongest association with the outcome is chosen.

Overall accuracy of the tree was calculated as the proportion of correctly classified patients out of the total number of patients included. A patient was considered correctly classified if the diagnosis assigned to their terminal node matched the final diagnosis established at the DSGZ. To evaluate classification performance for each vestibular disorder, we additionally reported sensitivity (SEN) and specificity (SPEC) with reference to VM.

For all statistic tests, a two-sided p-value below 0.05 was considered significant. Conditional inference trees were calculated with the “partykit” package [33]. All statistic calculations were performed with R (version 4.1.2).

## Results

We included a total of 290 patients with a mean age of 46.8 years (standard deviation SD = 14.4), 64.8% female. Of the 290 included patients 188 had VM (64.8%), 88 MD (30.3%), and 14 a mixed syndrome (4.8%). Patients with mixed syndrome were excluded from further analysis. 47% of patients had vertigo or dizziness for less than two years. For more details see Table 2.

**Table 2:** Description of the study sample separated for the two diagnoses vestibular migraine (VM) and Menière’s disease (MD)

Variable	All	VM	MD	*p*-value^b^
Sample size	276	188	88	-
Gender = female	96 (35%)	135 (72%)	45 (51%)	0.0013
Age	46.9 (SD = 14.4)	43.4 (SD = 13.6)	54.3 (SD = 13.1)	< 0.0001
Age of manifestation	42.9 (SD = 14.2)	39.6 (SD = 13.5)	49.9 (SD = 13.0)	< 0.0001
Duration of the disease	3.9 (SD = 6.0)	3.8 (SD = 6.2)	4.1 (SD = 5.8)	0.7032
DHI (Total Score)	42.4 (SD = 20.2)	41.6 (SD = 19.5)	44.2 (SD = 21.8)	0.3599
Attack characteristics				
Type of Vertigo Attacks				
Rotational	180 (66%)	108 (58%)	72 (83%)	< 0.0001
Swaying	128 (47%)	96 (51%)	32 (37%)	0.0342
Dizziness	20 (7%)	18 (10%)	2 (2%)	0.0547
Attack frequency (once or several times per …)				0.8084
Day	50 (22%)	37 (24%)	13 (19%)	
Week	62 (28%)	44 (28%)	18 (26%)	
Month	73 (32%)	49 (32%)	24 (34%)	
Year	24 (11%)	15 (10%)	9 (13%)	
Less 1 per year	16 (7%)	10 (6%)	6 (9%)	
Attack length				0.0005
Less 2 min	24 (9%)	20 (11%)	4 (5%)	
2 to 20 min	32 (12%)	26 (14%)	6 (7%)	
20 min to 1 h	30 (11%)	16 (9%)	14 (16%)	
1 h to 12 h	131 (48%)	76 (41%)	55 (62%)	
> 12 h	9 (3%)	6 (3%)	3 (3%)	
Several days	47 (17%)	41 (22%)	6 (7%)	
Selected self-reported subjective clinical signs and symptoms				
Vomiting	117 (42%)	54 (29%)	63 (72%)	< 0.0001
Ocular motor disturbance	74 (27%)	43 (23%)	31 (35%)	0.0441
Nausea	187 (68%)	119 (63%)	68 (77%)	0.0295
Gait instability	196 (71%)	126 (67%)	70 (80%)	0.0461
Headache	137 (50%)	113 (60%)	24 (27%)	< 0.0001
Photophobia	119 (43%)	91 (48%)	28 (32%)	0.0138
Paresthesia	60 (22%)	49 (26%)	11 (12%)	0.0169
Hearing problems	74 (27%)	24 (13%)	50 (57%)	< 0.0001
Tinnitus	126 (46%)	65 (35%)	61 (69%)	< 0.0001
Neck pain	98 (36%)	75 (40%)	23 (26%)	0.0365
Lifestyle				
Physically active	153 (56%)	100 (53%)	53 (61%)	0.3057
Ever smoked	112 (49%)	68 (45%)	44 (57%)	0.1224
Alcohol consumption = yes	116 (42%)	80 (43%)	36 (41%)	0.8989
Vestibular testing results				
Side difference on vestibular testing in %	23.8 (SD = 21.2)	19.4 (SD = 17.71)	34.0 (SD = 24.89)	< 0.0001
Side difference > 25%	79 (34%)	41 (25%)	38 (54%)	< 0.0001
Median Gain in vHIT^c^	0.86 (SD = 0.15)	0.89 (SD = 0.12)	0.80 (SD = 0.20)	< 0.0001
Median Gain < 0.70	26 (10%)	10 (6%)	16 (19%)	0.0015
Caloric-vHIT^c^ dissociation	66 (28%)	37 (23%)	29 (40%)	0.0112
Side of Gain Loss				0.0008
Normal	240 (90%)	171 (94%)	69 (81%)	
Unilateral	16 (6%)	8 (4%)	8 (9%)	
Bilateral	10 (4%)	2 (1%)	8 (9%)	
Refixation saccades	35 (16%)	11 (7%)	24 (34%)	< 0.0001
Subjective visual vertical	40 (14%)	18 (10%)	22 (25%)	0.0013
Ocular motor findings				
Spontaneous nystagmus	4 (1%)	3 (2%)	1 (1%)	1
Provocation-Induced Nystagmus	33 (12%)	16 (9%)	17 (19%)	0.0173
Ocular torsion	6 (2%)	3 (2%)	3 (3%)	0.6032
Gaze Holding	20 (7%)	11 (6%)	9 (10%)	0.2902
Saccadic Smooth Pursuit	144 (52%)	98 (52%)	46 (52%)	1
Downbeat Nystagmus	5 (2%)	5 (3%)	0 (0%)	0.2893
Upbeat Nystagmus	12 (4%)	8 (4%)	4 (5%)	1
Fixation-suppressed Nystagmus	16 (6%)	12 (6%)	4 (5%)	0.7396

We present absolute and relative frequencies as percentages for categorical variables and mean and standard deviation (SD) for continuous variables. All vestibular tests and ocular motor findings were performed during the asymptomatic interval at the time of presentation

^a^DHI: Dizziness Handicap Inventory

^b^t-Test for independent samples or Pearson’s Chi-squared test

^c^Video Head Impulse Test

### Vestibular function and audiometric test data

Vestibular function tests revealed significant differences between the groups with MD patients demonstrating higher side differences in caloric testing compared to VM patients (means 34.0% and 19.4%, *p* < 0.0001). Median gain was lower in MD (mean 0.80) relative to VM (mean 0.89, *p* < 0.0001) with pathological gain values observed in 19% of MD patients and 6% of VM patients (*p* = 0.0015). Caloric-vHIT dissociation was observed in 40% of MD patients and in 23% of VM patients (*p* = 0.0112). Based on side-specific vHIT gain values, 74% of all patients showed normal vestibular function, 17% had unilateral, and 10% had bilateral gain loss. In the VM group, 80% were classified as normal, 14% as unilateral, and 6% as bilateral while 61% of MD patients had normal gain, 21% showed unilateral and 18% bilateral impairment (*p* = 0.0022). Refixation saccades were also more frequent in MD (34%) compared to VM (7%; *p* < 0.0001). Abnormal subjective visual vertical was significantly more frequent in MD patients (25%) compared to VM (10%, *p* = 0.0013). Additionally, provocation-induced nystagmus occurred more often in MD (19% vs. 9%, *p* = 0.0173), while other orthoptic findings showed no significant group differences.

Pathological audiometry was found in 78% of MD patients, with 15% showing normal results and 7% missing data. In the VM group, 16% had pathological findings, 31% had normal results, and 52% lacked audiometric data. Due to the high proportion of missing data in the VM group compared to the MD group (52% vs. 7%), audiometric findings were not included in further analyses. This imbalance limited their clinical usefulness for differentiating between VM and MD.

### Medical history

VM patients were more likely to be female (VM 72% vs. MD 51%, *p* = 0.0013) and younger (VM: mean age 43.4 years, SD = 13.6 vs. MD: mean age 54.3 years, SD = 13.1). VM manifested earlier in life with an average age at first manifestation of 39.6 years vs. 49.9 years in patients with MD (*p* < 0.0001). The type of vertigo attacks varied, with rotational vertigo being more common in MD (83%) than VM (58%) with *p* < 0.0001 and swaying (51% vs. 37%) and dizziness (10% vs. 2%) being more frequent in VM.

Regarding accompanying symptoms, vomiting (72% vs. 29%, *p* < 0.0001), nausea (77% vs. 63%, *p* = 0.0295), gait instability (80% vs. 67%, *p* = 0.0461), and self-reported subjective ocular motor disturbance (35% vs. 23%, *p* = 0.0441) were significantly more common in MD patients. In contrast, headache (60% in VM vs. 27% in MD, *p* < 0.0001), photophobia (48% vs. 32%, *p* = 0.0138), and paresthesia (26% vs. 12%, *p* = 0.0169) were more frequently reported by VM patients. Hearing problems and tinnitus were more prevalent in MD (57% vs. 13%, *p* < 0.0001 and 69% vs. 35%, *p* < 0.0001), while neck pain occurred more often in VM (40% vs. 26%, *p* = 0.0365).

No significant differences were observed between the MD and VM in terms of impact on daily life, as measured by the DHI.

More details on patients’ characteristics are shown in Table 2 and in the electronic appendix, Table 1.

### Model to differentiate VM and MD

**Fig. 1:**
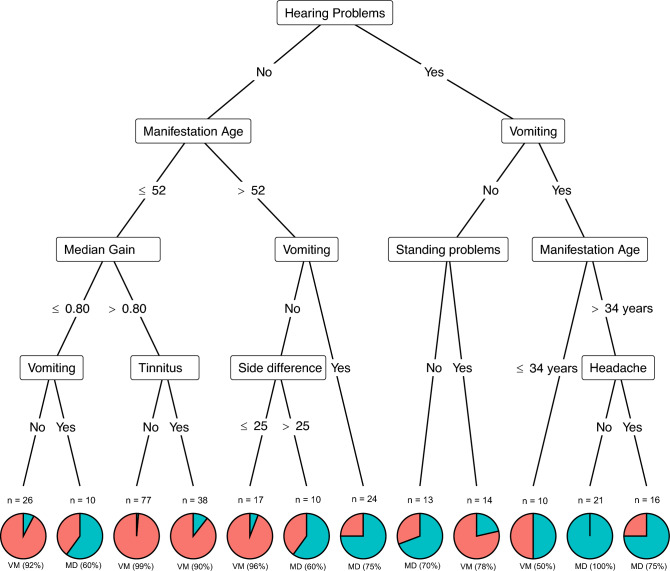
Result of the Conditional Inference Tree to distinguish between vestibular migraine (VM) and Menière’s disease (MD). Side difference refers to the side difference in caloric testing and the median gain to video head impulse testing

Seven variables turned out as important to differentiate VM and MD: presence of hearing problems, age at manifestation of the disease, vomiting, side difference in vestibular testing, standing problems, tinnitus, and headache. The resulting tree is shown in Fig. 1 with each node presenting the name of the splitting variable and each branch is labeled with the splitting criterion.

To give an example for interpretation: Among individuals without hearing problems and an age of manifestation below 52 years, only one subgroup remained suggestive of MD: those with a median vHIT gain below 0.80 and vomiting as a reported symptom. This tree had an overall accuracy of 86%, a sensitivity of 88%, and a specificity of 82%, i.e., the tree algorithm correctly classified 88% of patients with VM, and 82% of MD patients.

### Indicators of disagreement of clinical expert evaluation and ICVD criteria for diagnosis of VM

Table 3 gives an overview of the agreement of the clinical evaluation and the evaluation following the ICVD criteria. A total of 66 patients were clinically diagnosed with VM based on the overall judgement of symptoms, test results and features, but did not fulfill the ICVD criteria for VM. We refer to this constellation as patients with ‘suspected VM’.

**Table 3:** Agreement of the clinical evaluation and the evaluation following the Barany criteria

		Clinical evaluation		
		Suspected VM	Probable VM	Definite VM
ICVD Criteria	No VM	66	0	0
	Probable VM	0	86	9
	Definite VM	0	7	20

Patients with suspected VM had a shorter disease duration (2.2 vs. 4.7 years, *p* = 0.0091) compared to those meeting at least probable VM criteria by both assessments. They exhibited a higher prevalence of dizziness (17% vs. 6%, *p* = 0.0314) and lower frequency of vomiting (17% vs. 35%, *p* = 0.0118), light sensitivity (33% vs. 57%, *p* = 0.0039), sensation of a prickling feeling (15% vs. 32%, *p* = 0.0197) and ear-related symptoms, including ear pressure (18% vs. 38%, *p* = 0.0093) and tinnitus (23% vs. 41%, *p* = 0.0187). A table summarizing the key features of patients with discordant diagnoses between the clinical judgement and ICVD-based criteria can be found in the electronic appendix Table 2.

### Reasons for disagreement

To identify the most critical factors contributing to diagnostic discrepancies between clinical judgment and ICVD criteria in suspected VM, we systematically analyzed the underlying reasons for disagreement as documented by experienced clinicians.

The most frequent reason for discrepancy was atypical headache characteristics (*n* = 41), including occipital, frontal, holocephalic, or non-pulsating headache types, which did not conform to the ICVD definition of migraine. Attack characteristics inconsistent with ICVD criteria (*n* = 33) also played a major role, particularly when the total number of attacks was lower than the required five or when the duration of episodes fell outside the defined time range of 5 min to 72 h. Additional discrepancies arose from accompanying symptoms (*n* = 29) that are suggestive of VM but not explicitly addressed in the ICVD criteria, such as visual disturbances, perioral or facial paresthesia, or the need for rest during or following an attack.

While atypical headache types, non-conforming attack characteristics, or specific accompanying symptoms were in some cases considered sufficient alone to support a clinical diagnosis of suspected VM as primary diagnostic indicators, other factors rated as supportive information appeared only in conjunction with this core symptomatology. These included subtle central ocular motor findings (e.g., central positional nystagmus, gaze-evoked nystagmus, saccadic smooth pursuit, or minor deviations in subjective visual vertical), specific triggers (e.g., sleep deprivation, hormonal fluctuations, stress), comorbid conditions commonly associated with VM (e.g., motion sickness), and a positive family history. Although central ocular motor findings were reported with similar frequency in both VM and MD groups (Table 2), in suspected VM cases they were observed exclusively in combination with other features. The most common combinations involved headache characteristics in conjunction with attack features and accompanying symptoms, as well as various pairings with ocular motor findings. An overview of the most frequently cited reasons for diagnostic disagreement, i.e. suspected VM, is provided in Table 4.

**Table 4:** Reasons for disagreement of the clinical evaluation and the evaluation according to ICVD criteria of the 66 patients with suspected VM

Reason for disagreement of ICVD and clinical expert evaluation in VM patients	Number of Cases
Attack characteristics	33
Headache characteristics	41
Accompanying symptoms	29
Trigger	9
Comorbidities	5
Central ocular motor findings	
Gaze-evoked nystagmus	3
Provocation-induced nystagmus	1
Upbeat nystagmus	1
Downbeat nystagmus	2
Central positional nystagmus	2
Saccadic smooth pursuit	11
SVV deviation	9
Positive family history	14

## Discussion

The primary objective of this study was to characterize the differences between patients with Menière’s disease (MD) and vestibular migraine (VM) presenting at a specialized tertiary dizziness center and to identify factors that can effectively differentiate these two conditions. Conditional inference tree algorithm identified hearing problems, age at manifestation, vomiting, caloric asymmetry, standing problems, tinnitus, and headache as key differentiators yielding a model with 86% accuracy. Additionally, the study investigated the reasons for discrepancies between clinical expert evaluations and assessments based on ICVD criteria. Most often reported reasons were atypical headache types, non-conforming attack characteristics, or specific accompanying symptoms.

### Differentiation of VM and MD

An important problem in the diagnostic classification of VM is the differentiation from other episodic vestibular syndromes, especially from MD. Factors that complicate the differentiation of VM and MD are the common absence of headache with the attacks in VM or of ear and hearing symptoms in MD attacks, which is especially frequent at the beginning of these disorders [34]. A substantial proportion of patients with MD also report migrainous symptoms, contributing to diagnostic uncertainty. For example, Radtke et al. (2002) reported that 56% of patients with MD had a lifetime history of migraine, compared to 25% in age-matched controls. Furthermore, 45% of MD patients consistently experienced at least one migrainous symptom (e.g., headache, photophobia, aura) during vertigo attacks [17]. These findings underscore the considerable symptomatic overlap between VM and MD, even though formal diagnostic criteria may not be fulfilled simultaneously. Other studies have similarly highlighted this overlap [15, 16, 18].

Regarding the differences between the two entities, our study showed that VM is more prevalent in women and tends to manifest at a younger age than MD, which confirmed findings in literature [1, 35–38]. Attack characteristics differed significantly, with VM patients more frequently experiencing sensations of swaying and dizziness, while rotational vertigo was more common in MD. Vestibular function testing showed greater caloric side difference and lower vHIT gain, and a higher percentage of unilaterally pathologic vHIT gain in patients with MD, consistent with previous findings [13, 39]. Saccadic smooth pursuit was observed in 52% of patients in both groups. This high number likely reflects non-specific central susceptibility, age-related factors, or examiner variability, and its diagnostic value appears limited when considered in isolation.

In addition to caloric side difference the classification model identified vHIT gain as an important factor to distinguish between VM and MD, along with hearing problems, tinnitus, standing difficulties, vomiting, age at manifestation, and headache. However, these clinical features did not form entirely homogenous diagnostic groups as the allocation of a single symptom to diagnostic groups is not possible as shown before [15]. For example, the absence of headache in patients with hearing problems, vomiting, and age at manifestation over 34 years strongly suggested MD, while the presence of headache alone was not sufficient to classify patients exclusively as VM. Notably, we did not confirm headache as a hallmark feature of VM, as previously reported [40]. Other studies also have shown that approximately 30% to 50% of VM patients do not experience headaches associated with vertigo episodes [41, 42].

Side difference in caloric testing turned out as important in the differentiation between the two diagnostic groups. For instance, in individuals without hearing problems, manifestation age above 52 years and no vomiting a side difference below 25% serves as a strong indicator for vestibular migraine (96%) and a side difference above 25% identifies a heterogenous group, with over 60% meeting criteria for MD. This finding aligns with previous literature, which similarly emphasizes the diagnostic utility of caloric testing to distinguish between MD and VM [13, 39].

Caloric–vHIT dissociation was significantly more common in MD than in VM, consistent with previous findings suggesting that a mismatch between low- and high-frequency canal function is characteristic of MD [29]. This pattern likely reflects differential involvement of vestibular structures and highlights the added diagnostic value of combining caloric testing with vHIT.

Few studies have employed transparent learning methods, such as classification and regression trees (CART), for vestibular disorder differentiation. One former study also applied classification trees and identified hearing problems, disease duration, attack frequency, severity of rotational vertigo, onset and type of hearing loss, and prior head trauma as significant diagnostic variables [43]. While these findings largely align with our results, certain factors, such as head trauma, were not examined in our study. Additionally, that study aimed to develop an algorithm for classifying a broader range of vestibular disorders, whereas our focus was specifically on distinguishing VM from MD. A recent study applied machine learning to classify recurrent spontaneous vertigo but included only patients meeting ICVD criteria for VM and MD [44]. Their models, while more effective, relied on black-box algorithms such as random forest and XGBoost, which offer limited interpretability. In contrast, our approach provides a transparent, clinically intuitive model.

### Suspected VM—indicators of disagreement of clinical expert evaluation and ICVD criteria

VM is simple to diagnose if (1) the attacks are always or mostly followed by migraine-type headache and (2) the patient has a positive personal history of a migraine as defined by the criteria of the International Headache Classification. Establishing the diagnosis, however, may prove difficult, if the symptom features are more ambiguous. VM often is considered the chameleon among the episodic vestibular syndromes. There are several reasons for this: headache is absent in about 30% of patients, the characteristics of vertigo may be diverse and may vary (also in severity), vertigo attacks may last from seconds to several days, and MD may coincide with VM [41, 45–51].

Despite the current plethora of literature on VM several questions remain unsolved. The occurrence of symptoms such as hypersensitivity to light and sound, a need for quiet environments, tiredness after the attack, an urge to urinate, and a pronounced susceptibility to motion sickness may support the clinical diagnosis of VM. However, these ‘soft’ criteria are currently not included in the ICVD diagnostic framework. Nevertheless, these clinical signs are often decisive in cases where the diagnosis of VM is challenging. In our study we had classified 66 patients as ‘suspected VM’ according to our clinical assessment although they did not fulfill ICVD criteria, underscoring the limitations of current diagnostic guidelines. This was primarily due to atypical symptoms, deviating attack duration, central ocular motor abnormalities, and additional VM-related triggers. In detail these were bilateral or occipital headache localization, accompanying symptoms such as a need for rest or paresthesia in the facial area, triggers such as stress, lack of sleep or hormone association, and a positive family history being particularly indicative.

In these suspected patients, we observed subtle central ocular motor signs such as saccadic smooth pursuit or gaze-evoked nystagmus. These ocular motor abnormalities, even during attack-free intervals, have been repeatedly described in the literature [47, 52–55]. They may be more severe during the attack and are often associated with postural instability. An important distinguishing feature from structurally caused central vestibular syndromes, e.g., by a cerebrovascular event, is that in the case of VM there is usually no additional clinical evidence of a central vestibular damage [56–58].

To be able to provide patients with ‘suspected VM’ with the appropriate therapy, attention should be paid to soft clinical and ocular motor signs indicative for VM, which can of course also be present in combination. This includes the fact that, in line to our clinical experience, headache in VM does not necessarily have to be on one side, but is often localized bilateral occipital, frontal or holocephalical and can have a pressing character.

### Limitations

The diagnostic workup in our cohort followed a pragmatic clinical approach, which led to a lack of standardized audiometric testing, particularly among VM patients who often did not report hearing-related symptoms. As a result, due to the limited availability of detailed audiometric data in many VM cases, we were unable to draw definitive conclusions regarding the role of audiometry in the classification of VM versus MD. Since VM is frequently diagnosed by excluding MD, this absence of audiometric data raises the possibility that some patients may have had undetected MD or may develop audiometric abnormalities over time, especially those classified as suspected VM [59]. Similarly, other neurophysiological modalities, such as vestibular evoked myogenic potentials (VEMPs), were not applied consistently across all subjects, given their limited diagnostic accuracy in differentiating between these conditions.

To minimize diagnostic ambiguity, we excluded patients with a mixed diagnosis of VM and MD. However, clinical overlap is well recognized and may even emerge over time, particularly in early-stage or atypical presentations. Recent studies suggest that migraine is more common in MD than previously assumed, and that some patients may eventually fulfill criteria for both conditions highlighting the need for longitudinal follow-up [60]. Overall, we believe that the path towards a more sensitive and clinically meaningful classification does not lie in increasingly fine-grained instrumental testing alone, but rather in a comprehensive and nuanced evaluation of the patient’s description of attack characteristics. Importantly, both VM and MD should be regarded not as clearly defined biological disease entities, but as conceptual constructs encompassing a spectrum of symptoms with heterogeneous and incompletely understood pathophysiological mechanisms.

With regard to the statistical analysis, conditional inference trees offer clear advantages in terms of interpretability but also present certain limitations. First, single-tree models generally underperform compared to more complex ensemble methods, such as random forests or gradient boosting, in terms of predictive accuracy. Studies on both real and simulated datasets suggest that the best single-tree models tend to have, on average, 10% lower accuracy than ensemble methods [61]. Second, tree-based models are sensitive to small variations in the dataset, which can result in considerable changes to the tree structure and splitting variables.

Despite these limitations, we consider conditional inference trees (CIT) to be well-suited for the aims of this study due to their transparency and alignment with clinical reasoning. Unlike black-box machine learning algorithms, tree-based models provide intuitive decision pathways that closely mirror clinical thinking, making them particularly valuable for structuring and interpreting diagnostic assessments and facilitate insight into the underlying decision logic, which is critical in complex diagnostic scenarios.

## Conclusion

Diagnosing VM is challenging due to its overlap with MD and limitations of current ICVD criteria. This study addresses both key challenges: reliably differentiating VM from MD, and identifying clinically relevant cases of VM that do not meet the formal ICVD criteria.

The differentiation between vestibular migraine and Menière’s disease was best achieved using seven key clinical variables: hearing problems, age at manifestation, vomiting, caloric side difference, gain values, standing problems, tinnitus, and headache. While our identified model demonstrated high accuracy in cross-sectional data, its long-term validity remains to be established. Prospective follow-up studies will be essential to confirm whether these patients develop stable clinical patterns or transition toward alternative diagnoses. In cases of’suspected VM’, not meeting ICVD criteria, discrepancies were most often linked to short attack duration, migraine symptoms not included in the ICVD (e.g., paresthesia), family history and subtle central ocular motor findings, including the presence of gaze-evoked or positional nystagmus or saccadic smooth pursuit.

These results highlight the importance of integrating structured clinical reasoning with nuanced symptom evaluation. Our findings support a more profound, clinically grounded approach to improve diagnostic accuracy and patient care in specialized settings.

## Data Availability

An anonymized version of the dataset used and analyzed during the current study is available from the corresponding author upon reasonable request.
